# Nonlinear Response of a Polycarbonate in Post-Yield Cyclic Tests

**DOI:** 10.3390/polym17111535

**Published:** 2025-05-31

**Authors:** David Trejo Carrillo, Alberto Díaz Díaz

**Affiliations:** Centro de Investigación en Materiales Avanzados, S.C. (CIMAV), Av. Miguel de Cervantes #120, Complejo Industrial Chihuahua, Chihuahua C.P. 31136, Mexico; david.trejo@cimav.edu.mx

**Keywords:** polycarbonate, glassy state, nonlinear behavior, plastic dilatation, mechanical test

## Abstract

This paper aims to investigate the mechanical behavior of a polycarbonate through cyclic tensile, compression, and torsiontests atstrain rates that reduce viscous effects for this material. Measurements included axial and transverse strains for uniaxial tests and shear strains for torsion. Tensile tests exhibited nonlinear elasticity, ratcheting, and plasticity, accompanied by an increase in volumetric strain. Compression tests revealed nonlinear elasticity, with the surprising result of positive plastic axial and volumetric strains, accompanied by marginal transverse strains. Torsional tests showed an elastic but nonlinear relationship between shear stress and strain. In these latter tests, positive plastic volumetric strains were observed, which suggests that deviatoric stress can also induce volumetric plastic strains. These findings are of great importance for developing mathematical models of glassy amorphous polymers, and the observations contribute to understanding the complex behavior of such materials.

## 1. Introduction

A glassy state describes the condition of any amorphous polymer below its glass transition temperature (Tg). At room temperature, polycarbonate (PC) is in a glassy state. It possesses good mechanical properties which have been extensively investigated in various aspects, such as their dependence on strain rate and temperature [1,2,3,4], molecular weight [5], and physical aging [2,6], among others. Its properties make it suitable for various applications, although it exhibits a complex mechanical behavior. A non-crystalline structure characterizes this material by forming packing defects in a non-equilibrium state, directly influencing its properties [7].

Glassy amorphous polymers have free volume in their structure, although “free volume” is not uniquely defined. Static-free volume does not depend on temperature or time. It is related to the configuration of the chains, as space is created between them due to insufficient chain packing. Even if the chains were perfectly aligned, gaps would still exist between them. In contrast, dynamic-free volume results in the chains’ movement by thermal activation or for changes dependent on time [8,9]. The latter, known as excess free volume, occurs because these materials are out of thermodynamic equilibrium below Tg. Over time, the polymer chains relax towards equilibrium by local and segmental movement to eliminate this excess free volume, which gradually approaches equilibrium, in a process known as physical aging [10] that leads to changes in material properties [11,12,13]. In the case of PC, it has been observed that physical aging affects its mechanical properties [14,15] and reduces the free volume.

Mechanical loads on amorphous glassy polymers induce volume changes, as the free volume varies during deformation [16,17]. For example, Xie et al. [18] showed that the size of the existing microvoids is altered in PC specimens in tension and compression. While some studies suggest that mechanical deformation can mitigate the effects of physical aging, interpreting such results remains a topic of debate [19]. However, plastic strains significantly alter the thermodynamic state of the material [12,20] due to changes in the structure and, therefore, in the free volume.

Volumetric expansion has been observed in both tension and compression and is associated with inelastic deformation resulting from the hydrostatic component of the stress [21,22,23,24,25,26]. It is scarcely specified whether such inelastic deformation is related to time-dependent phenomena or plasticity, or what occurs in the transverse direction. Both experimental results and molecular simulations suggest that deformation in polymers is a complex process involving the simultaneous occurrence of several mechanisms that cause changes in volumetric deformation. For instance, it includes an increase in free volume, molecular reorganization, and orientation [26,27,28,29], as well as intramolecular and intermolecular interactions [30,31]. Studies on PC have shown that plastic deformation leads to volumetric changes [32,33].

Traditionally, the stress–strain behavior of glassy amorphous polymers is considered linear elastic at low strain levels, with deviations from linearity attributed to viscous effects as strain increases [34]. However, some studies indicate a more complex response even at low strain levels, suggesting nonlinear elastic behavior, such as hypoelasticity and the onset of plastic phenomena at relatively small strains. For instance, Rey Calderón and Díaz Díaz [35] used polycarbonate specimens and observed hypoelastic behavior in compressive tests. Similarly, Quinson et al. [36] investigated polycarbonate in compression and identified the inelastic development. More recently, Bargagallo et al. [37] also analyzed the mechanical response of polycarbonate under tensile loading, showing deviations from purely elastic behavior at low strains. Unloading during a mechanical test provides valuable insight into the material’s behavior, as strain can be divided into elastic and inelastic components (viscous or plastic). Elastic deformation is recovered instantly upon unloading, while viscous deformation recovers over time. In contrast, plastic deformation remains permanently in the material [36].

In ref. [35], Rey Calderón and Díaz Díaz focused on the viscous phenomena in a Makrolon GP solid PC (1.200 g/cm^3^ density), usually used for its high impact resistance and dimensional stability. They performed creep and monotonic uniaxial tests at various temperatures and strain rates. The authors observed an asymmetry effect on the mechanical behavior and revealed a nonlinear master transverse strain/axial strain curve valid for monotonic tests. In compression, a nonlinear elastic behavior was revealed despite the axial strains being smaller than 6%. Owing to a zero-stress recovery stage at the end of the creep tests, the authors detected minor plastic strains compared to the viscoelastic ones.

The present work investigates nonlinear phenomena (plasticity and nonlinear elasticity) and volumetric strains in PC specimens within a small-strain framework (strains are smaller than 10%). Unlike the approach in [35], to promote the occurrence of plastic rather than viscous strains, we employ 20 times higher strain rates and a lower-strength commercially available polycarbonate (1.186 g/cm^3^ density). Tensile, compression, and torsional cyclic tests were conducted to determine permanent strains and the effects of deviatoric stresses. Special attention was given to residual plastic deformation and its associated volumetric changes across all tests.

## 2. Materials and Methods

**Figure 1:**
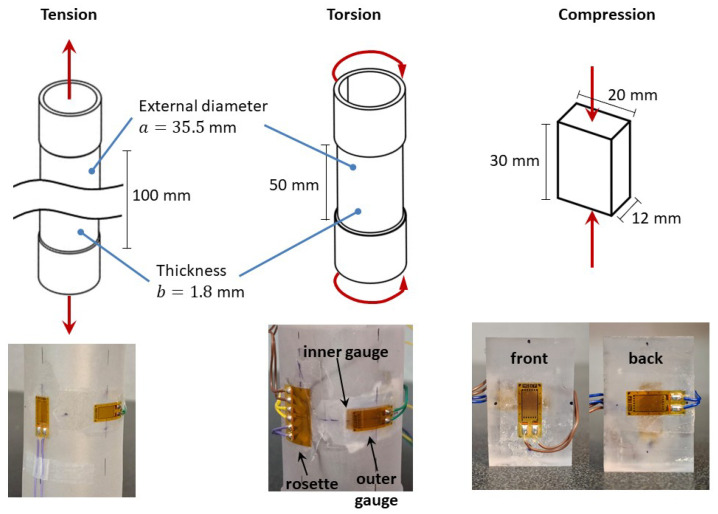
Dimensions of specimens and location of strain gauges.

A PC with an average molecular weight of 55 kg/mol (*Mw*), determined via gel permeation chromatography, a Tg of 145 °C, as measured by differential scanning calorimetry, and a density of 1.186 g/cm^3^ was used. The material is commercially available in tubes and is primarily employed in lighting fixtures and chemical handling due to its optical clarity and chemical stability. In Figure 1, the geometries of the specimens used are shown. For torsional tests, tubular specimens were cut and machined, obtaining an external diameter *a* of 35.5 mm and a thickness *b* of 1.8 mm to ensure a quasi-uniform shear stress state. No axial load was applied in torsional tests. The shear stress is related to the torque *T* as follows:

(1) $$\tau =T{\left[\pi \left(\frac{a^{4}}{32}-\frac{{\left(a-2b\right)}^{4}}{32}\right)\right]}^{-1}\frac{a}{2}.$$

The tubular specimens were also subjected to tension testing. For compressive tests and to avoid buckling, the tubes were transformed into plates by applying a 1.5 MPa pressure and a 220 °C temperature. To verify that this transformation into plates did not alter the material properties, tensile specimens obtained from the plates were tested, and their behavior was the same as that of the tubular ones. Compression specimens had a cross-section of 12 mm × 20 mm and a height of 30 mm. Before testing, all the specimens (for tension, compression, and torsion) were subjected to thermal rejuvenation by heating to 120 °C for 24 h, followed by heating to 155 °C for another 24 h, and then cooling to room temperature [35].

As shown in Figure 1, strain measurement was performed using strain gauges. A National Instruments Data Acquisition System was used to record the strains; this device is capable of measuring strains on the order of $10^{-6}$; however, due to its sensitivity to noise and vibrations, the reported results are limited to a precision of 10^−5^. In uniaxial tests, axial and transverse strains ($\varepsilon_{a}$ and $\varepsilon_{t}$, respectively) were measured using uniaxial strain gauges ( CEA-06-240UZ-120 model, Micro-Measurements, Wendell, NC, USA) located at the middle of the specimen. One can then deduce the volumetric strains $\varepsilon_{v}=\varepsilon_{a}+2\varepsilon_{t}$. In torsional tests, the principal strains $\varepsilon_{2}$ and $\varepsilon_{3}$ on the outer surface of the tube were measured with a strain gauge rosette (CEA-06-120CZ-120 model, Micro-Measurements); thus, the shear strain was deduced with the following formula: $\gamma =\varepsilon_{2}-\varepsilon_{3}$. The torsion specimen’s internal and external circumferential strains were also measured with uniaxial strain gauges (CEA-06-240UZ-120 model, Micro-Measurements) on its inner and outer surfaces. These measurements allowed the calculation of diameter changes and, subsequently, the thickness strain using the following expression:

(2) $$\varepsilon_{1}=\frac{a\varepsilon_{e}-\left(a-2b\right)\varepsilon_{i}}{2b},$$

where *a* is the external diameter, *b* the thickness, $\varepsilon_{e}$ is the external circumferential strain and $\varepsilon_{i}$ is the internal circumferential strain. This allowed the calculation of the volumetric strain during the torsional test:

(3) $$\varepsilon_{v}=\varepsilon_{1}+\varepsilon_{2}+\varepsilon_{3}.$$

**Figure 2:**
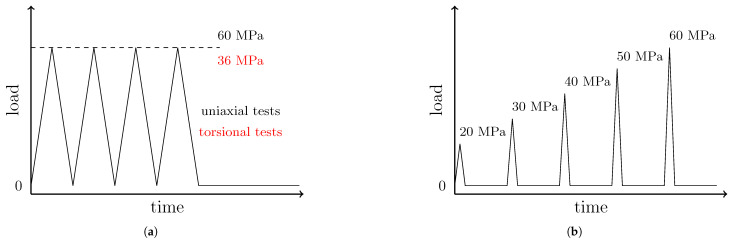
Diagrams of the applied load against time for type 1 tests (**a**) and type 2 tests (**b**).

Two types of cyclic tests were conducted at room temperature, well below the glass transition temperature. As shown in Figure 2a, the type 1 test consisted of four load-unload cycles with a fixed stress amplitude and a recovery time of 300 s at a quasi-load-free state after the last cycle to check for viscous contribution: 60 MPa for tensile and compression with a strain rate of 3.5%/s, and 36 MPa for torsion, with a shear strain rate of 5.5%/s. The stress levels ensure a post-yield state and an equivalent deviatoric stress (Von Mises stress). The aforementioned strain rates were selected to ensure that viscous strains are negligible compared to plastic strains (this will be confirmed in Section 3).

**Figure 3:**
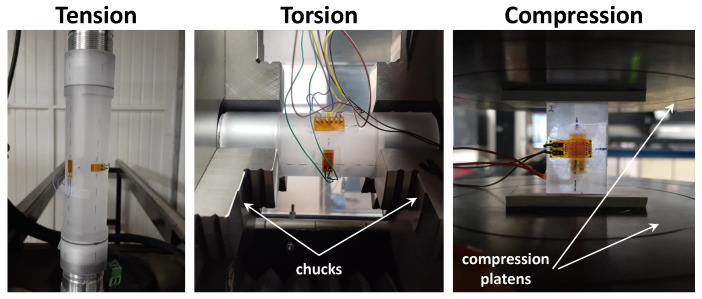
Specimens mounted on the universal testing machines.

The type 2 test is illustrated in Figure 2b; load-unload cycles were applied with increasing load levels (20, 30, 40, 50, and 60 MPa) for tension and compression, with a 60 s recovery between cycles to monitor the viscous contribution. In the loading and unloading stages, the strain rate was 3.5%/s. All tests were conducted on electromechanical machines: Instron 3318 and 55MT2 models for uniaxial and torsional tests, respectively (Instron, Norwood, MA, USA). As a result, the previously defined maximum stress values were sometimes exceeded due to device inertia and control issues at relatively high strain rates for these machines, which are mainly devoted to quasi-static tests. In Figure 3, the specimens are shown mounted on the universal testing machines.

## 3. Results

Three samples (A, B, and C) were tested for each type of test.

### 3.1. Tensile Tests

**Figure 4:**
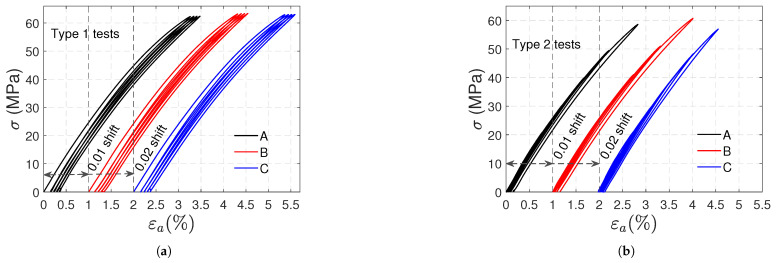
Artificially shifted stress vs. axial strain curves for type 1 (**a**) and type 2 (**b**) tensile tests.

In Figure 4, the stress (σ) vs. axial strain ($\epsilon_{a}$) curves obtained in these tests are shown. Figure 4a,b corresponds to type 1 and type 2 tests, respectively; notice that two artificial horizontal shifts (0.01 and 0.02 for samples B and C, respectively) were used to distinguish the curves of each sample. A nonlinear behavior is exhibited in the loading and unloading paths. In addition, residual strains remain at the moment of unloading; these strains increase in successive cycles, and in type 2 tests, the increment is also due to the higher stress level reached after each cycle. In type 1 tests, ratcheting is revealed: a shift of the stress–strain hysteresis loops along the strain axis provoked by successive loading cycles.

**Figure 5:**
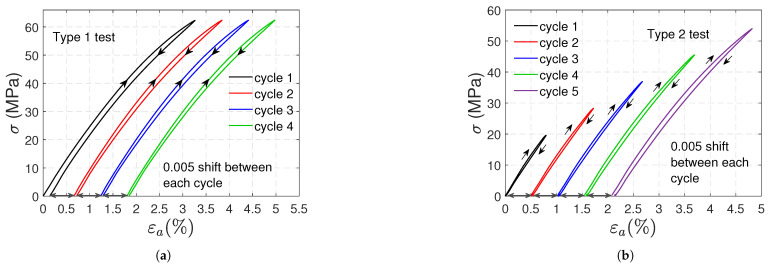
Artificially shifted stress vs. axial strain curves for type 1 (**a**) and type 2 (**b**) tensile tests (sample A).

To distinguish the loading and unloading paths within each cycle, in Figure 5a (Figure 5b), the stress–strain curve of sample A in type 1 (type 2) test was copied using a 0.005 horizontal shift after each cycle. It can be observed that the response is virtually linear below 20 MPa for both tests. For type 1 tests, the unloading paths are identical; the same can be said for the loading paths (except for the first cycle). For both types of tests, the slopes of the beginning of the loading paths and the end of the unloading paths are almost the same (2.7 GPa, i.e., the elastic modulus). The tangent modulus measured at the beginning of the unloading stage is smaller than the elastic modulus; it remains virtually constant after subsequent unloadings (1.52 GPa on average) for type 1 tests, and it slowly decreases with the number of cycles in type 2 tests.

**Figure 6:**
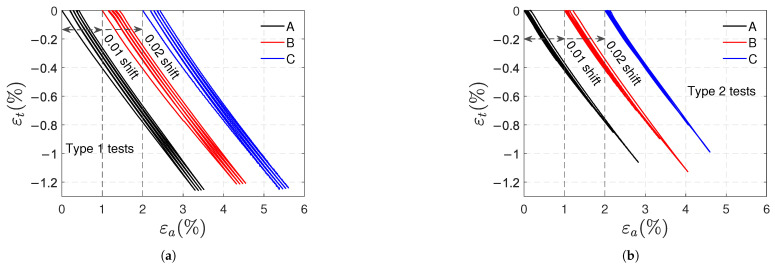
Artificially shifted transverse strain vs. axial strain curves for type 1 (**a**) and type 2 (**b**) tensile tests.

Figure 6a,b shows the transverse strain vs. axial strain curves of type 1 and type 2 tests, respectively. Once again, a horizontal shift was used to distinguish the curves of samples A, B, and C. Nonlinear behavior is observed in both loading and unloading paths, with an average Poisson’s ratio of 0.45, which corresponds to the absolute value of the slope of the curves at the beginning of the tests. In type 1 tests, the unloading paths have the same shape; the same can be said for the loading paths, except for the first cycle. Additionally, the absolute value of the slope of the curves at the beginning of unloading is, on average, 0.36. In type 2 tests, the absolute values of these slopes decrease in successive cycles because the load level increases.

**Figure 7:**
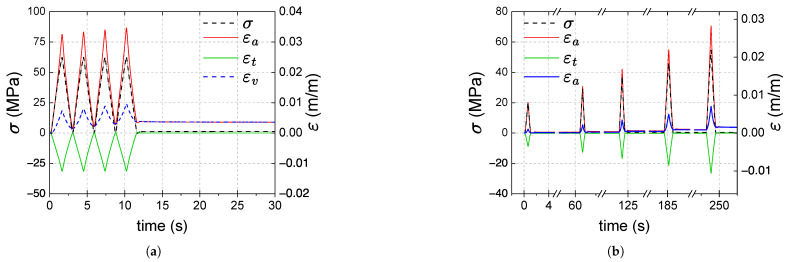
Stress and strains vs. time curves for type 1 (**a**) and type 2 (**b**) tensile tests.

**Figure 8:**
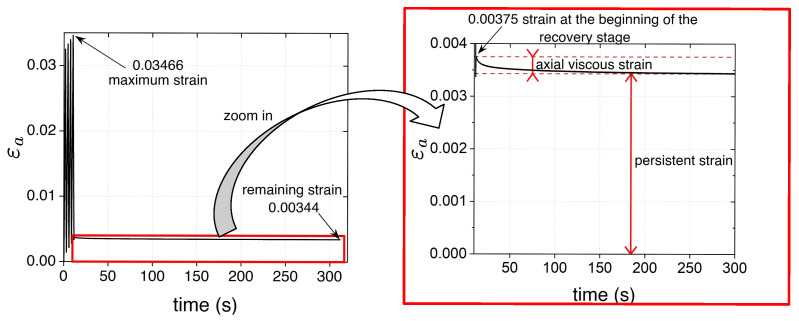
Axial strain vs. time curve during all the zero-stress recovery stage in a type 1 tensile test (sample A).

In Figure 7a,b, stress and strains (axial, transverse, and volumetric strains) are plotted against time for samples A in type 1 and type 2 tests, respectively. Similar curves were obtained for the other samples. In both types of tests, the transverse strain $\varepsilon_{t}$ is quasi-zero at the beginning and end of each cycle; no permanent transverse strains are observed. For type 1 tests:

- The transverse strains reached at the end of the loading stages are the same. Increasing axial and volumetric strains remain after each cycle, and permanent strains appear at the end of the test.
- The remaining $\varepsilon_{a}$ and $\varepsilon_{v}$ strains at the end of the test are on average $3.3\times 10^{-3}$ and $3.2\times 10^{-3}$; they correspond approximately to 9.4% and 31% of the maximum axial and volumetric strains reached during the test, respectively.
- a minimal viscous effect appears during the recovery stage: as shown in Figure 8, the axial strain at the beginning of the recovery stage is slightly higher than the persistent strain recorded at the end of the test, resembling a creep recovery-like evolution. The difference between the strains (axial viscous strain) is less than 1% of the maximum strain reached during the type 1 test.

In type 2 tests, viscous effects are also minimal, and residual axial and volumetric deformations are observed during the recovery stages; the remaining $\varepsilon_{a}$ and $\varepsilon_{v}$ strains increase progressively depending on the previously reached load level, and at the end of the test they stabilize at almost the same value $1.4\times 10^{-3}$.

### 3.2. Compression Tests

**Figure 9:**
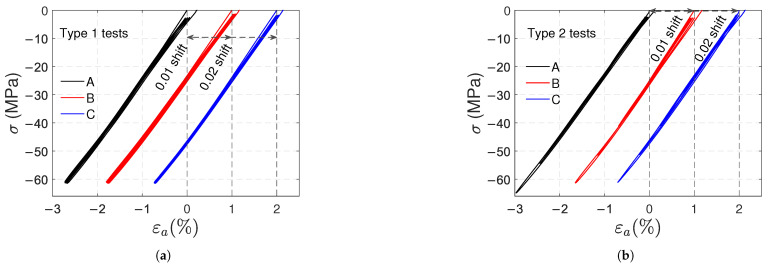
Artificially shifted stress vs. axial strain curves for type 1 (**a**) and type 2 (**b**) compressive tests.

Before presenting the results, it is worth noting that in these tests, to ensure the samples did not move due to the device’s inertia at the velocity used, it was not possible to reach the zero-stress level during the intermediate unloadings, during which a stress level of 2 MPa was attained. The zero-stress level was only achieved at the beginning and end of each test. In Figure 9a,b, the stress is plotted against the axial strain in type 1 and type 2 tests, respectively; the curves of samples B and C were shifted to the right (0.01 and 0.02 strain shifts were used, respectively) to ease visualizing them. The loading and unloading stages follow nonlinear paths, resulting in positive residual axial strains that persist during the recovery stage at the end of the test. This axial expansion is intriguing, given the applied compressive load. In Figure 9a,b, vertical dashed lines indicate the initial zero strain for each sample to highlight the axial expansions after the loading cycles. In the type 1 test, a slight ratcheting towards positive axial strains is observed.

**Figure 10:**
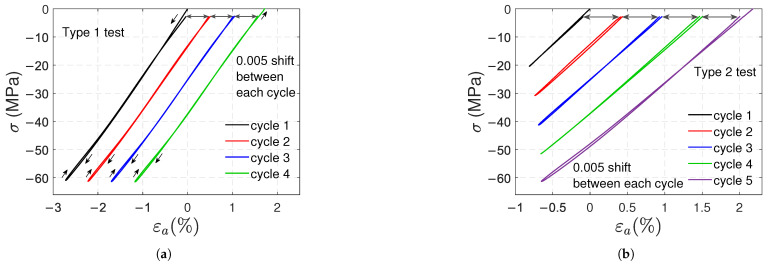
Artificially shifted stress vs. axial strain curves for type 1 (**a**) and type 2 (**b**) compressive tests (sample A).

To better illustrate the loading and unloading paths, the stress–strain curves of sample A in type 1 and type 2 tests were placed in Figure 10a,b by applying a 0.005 horizontal shift after each cycle, respectively. It can be observed that for compressive stresses below 26 MPa, the response is virtually linear for both tests. For type 1 tests, the loading paths are identical (except for the first loading); the same can be said for all the unloading paths. The elastic modulus (i.e., the slope at the beginning of the first loading) has an average value of 2.4 GPa; from the second loading cycle, the tangent modulus at the beginning of the loading path is slightly smaller than 2.4 GPa. The tangent modulus measured at the beginning of the unloading stage is smaller than the elastic modulus; it remains virtually constant after subsequent unloadings (1.97 GPa on average) for type 1 tests, and it slowly decreases with the number of cycles in type 2 tests.

**Figure 11:**
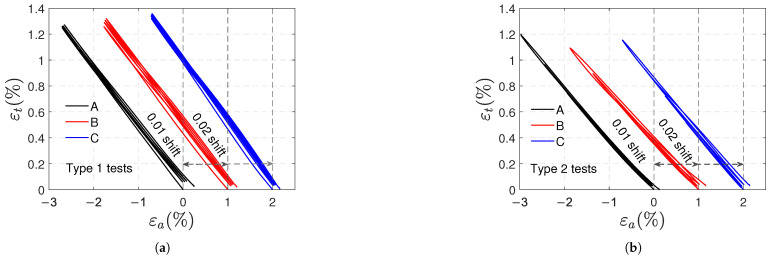
Artificially shifted transverse strain vs. axial strain curves for type 1 (**a**) and type 2 (**b**) compressive tests.

Figure 11a,b shows the curves of transverse strain against axial strain in type 1 and type 2 compressive tests, respectively. Let us define the tangent ratio as the absolute value of the slope of the line tangent to the curve. The obtained average of the tangent ratios at the beginning of the tests is 0.43, i.e., Poisson’s ratio. The tangent ratio at the initiation of a loading stage increases slightly after subsequent loading cycles. At the beginning of the unloading stages, the tangent ratio remains virtually constant (0.47) in type 1 tests, but slightly increases after subsequent loads in type 2 tests.

**Figure 12:**
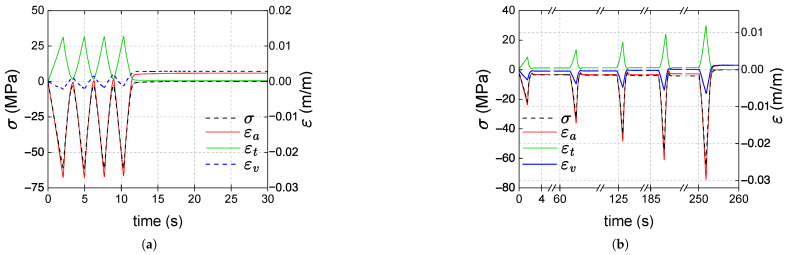
Stress and strains vs. time curves for type 1 (**a**) and type 2 (**b**) compressive tests.

Figure 12a,b shows the evolution of stress (σ) and strains ($\varepsilon_{a}$, $\varepsilon_{t}$ and $\varepsilon_{v}$) against time for samples A in type 1 and 2 tests, respectively. The curves of the other samples were similar. At the end of both tests, positive strains remain ($\varepsilon_{a}$, $\varepsilon_{t}$ and $\varepsilon_{v}$); the remaining transverse strain is smaller than axial and volumetric strains. For the type 1 tests, the remaining axial and volumetric strains are, on average, $1.9\times 10^{-3}$ and $2.2\times 10^{-3}$, respectively. For the type 2 test in Figure 12b, a small viscous effect appears at the end of the test. In this test, the remaining strains $\varepsilon_{a}$ and $\varepsilon_{v}$ at the end of each unloading tend to increase with the number of cycles; their values at the end of the test are $2.2\times 10^{-3}$ and $2.1\times 10^{-3}$, respectively.

### 3.3. Torsional Tests

**Figure 13:**
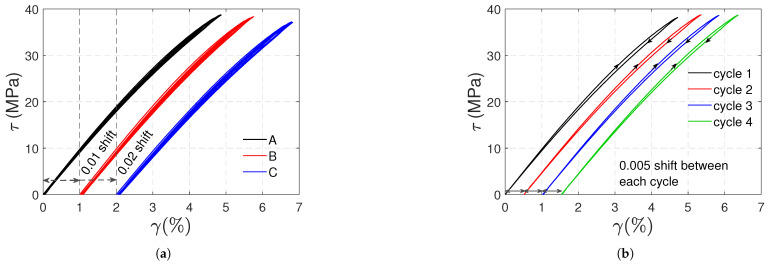
Artificially shifted shear stress vs. shear strain curves for type 1 torsional tests: samples A, B and C (**a**); cycles for sample A (**b**).

It is worth noting, as mentioned in Section 2, that due to inertia and control issues at the speed used, the shear stress of 36 MPa, which was set as the maximum value, was exceeded in some loading cycles. Similarly, instead of ending with zero shear stress, some unloadings reached negative shear stress. Figure 13a shows the shear stress (τ) vs. shear strain (γ) curves obtained in torsional tests; since no difference in the response of the three samples was observed, a 0.01 (0.02) horizontal shift was used to distinguish the curve of sample B (C) from that of sample A. The polycarbonate exhibits a nonlinear response in torsion. In Figure 13b, for sample A, the shear stress vs. strain curves of each cycle were shifted to ease the visualization of the loading and unloading paths; the response in each cycle is the same. The loading and unloading paths are nearly identical. A quasi-constant 0.97 GPa tangent modulus is obtained at the beginning of each loading stage.

**Figure 14:**
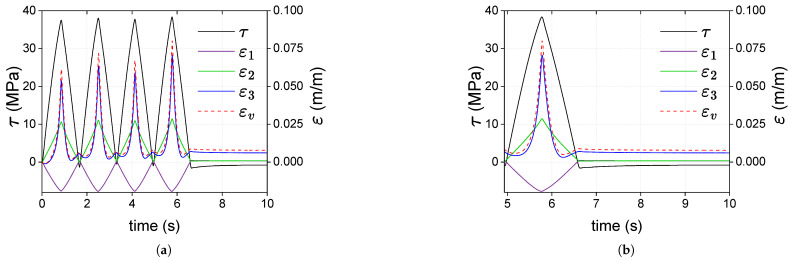
Shear stress (τ) and strains ($\varepsilon_{1}$, $\varepsilon_{2}$, $\varepsilon_{3}$ and $\varepsilon_{v}$) vs. time curves for sample A in torsion: (**a**) considering all cycles and a part of the recovery stage; (**b**) analysis of the last cycle and the recovery stage.

Figure 14a shows the curves of shear stress τ, principal strains ($\varepsilon_{1}$, $\varepsilon_{2}$, and $\varepsilon_{3}$) and volumetric strain $\varepsilon_{v}$ against time for the type 1 test on sample A. The response of PC exhibits asymmetry, with the maximum deformation in principal direction 2 reaching approximately $2.6\times 10^{-2}$, while the maximum absolute value in principal direction 3 is about $2.0\times 10^{-2}$. Additionally, as seen in the last cycle shown in Figure 14b, it is evident that the concavity of the strain curve for principal direction 2 differs from that of principal direction 3. At the beginning of the loading stages, $\varepsilon_{1}$ (the strain in the radial direction) and $\varepsilon_{v}$ remain virtually zero, but once the shear stress reaches 34 MPa, these strains increase significantly. Interestingly, at the end of the unloadings (zero shear stress), principal strains $\varepsilon_{2}$ and $\varepsilon_{3}$ are much smaller than $\varepsilon_{1}$. When the load is removed (zero shear stress), volumetric strains are almost the same as the $\varepsilon_{1}$, and a volume increase is then revealed. The values of $\varepsilon_{1}$ and $\varepsilon_{v}$ at the end of the test are $6.6\times 10^{-3}$ and $8.0\times 10^{-3}$; these strains remain virtually constant over time in the zero stress stage.

## 4. Discussion

The strain rates employed in our experiments lie at the upper bound of the quasi-static range typically reported in the literature [38]. Despite this, these strain rates are enough to reduce the viscous effects. As mentioned in Section 3, the zero stress recovery stage in the cyclic tests revealed that the permanent strains are significantly greater than the viscous ones. For example, in the case of a type 1 tensile test, as shown in Figure 8, the viscous strain recovered and the persistent strain are 0.034% and 0.34%, respectively. This might seem to contradict what Rey Calderón and Díaz Díaz observed at the end of uniaxial creep tests followed by a zero stress recovery stage [35]: viscous strains are recovered, leaving minimal persistent strains; for example, in a 50 MPa creep test with recovery at 50 °C, they reported a 0.23% viscous strain and a 0.06% permanent one. First, it is worth recalling that the polycarbonates studied are not the same: in [35], plates of Makrolon GP solid PC (1.2 g/cm^3^ density) intended for impact-resistant applications were used, whilst a tubular PC for optical and chemical handling applications is considered herein. Also, the strain rates applied are not the same: in [35], the highest strain rate used was 9%/min (the strain rate used in the loading/unloading stages in the creep test with recovery), i.e., approximately 23 times smaller than the present ones. The viscoelastic strains reported by Rey Calderón and Díaz Díaz are significantly greater than the viscous strains observed herein, mainly because the creep stages in [35] lasted 30 min and the monotonic tests took at least 30 s, whilst one cycle of the type 1 uniaxial tests performed herein took less than 3 s.

From this point forward, viscous strains are not considered in the discussion.

### 4.1. Plasticity and Nonlinear Elastic Behavior

The tests were performed in the post-yield regime. During the zero-stress stages after removing the loads, the persistent deformations (axial, transverse, and volumetric strains) that do not vary with time are considered plastic strains. This confirms the existence of plasticity, which is one factor contributing to the nonlinear behavior of PC. Positive plastic axial strains were obtained in tensile and compressive tests. No plastic shear strains were detected in torsional tests using tubular specimens; however, plastic strains were observed across the thickness. The plastic strains in all tests increased with the applied load and the number of cycles; consequently, ratcheting was observed in the uniaxial tests.

Nonlinear elasticity is also a phenomenon that contributes to polycarbonate’s nonlinear response. In the post-yield regime, the nonlinearities in the loading paths observed in the stress–strain curves definitely involve plasticity. Nevertheless, the nonlinearity of the unloading paths is more likely due to nonlinear elasticity. In Figure 5 and Figure 10, the loading and unloading paths are very similar, except for a slight and gradual shift caused by plasticity. Figure 13 demonstrates a nonlinear elastic relation between shear stress and strain in torsional tests without plastic shear strains.

### 4.2. Isotropy and Asymmetric Material Behavior

In the cyclic tests shown in this paper, Young’s modulus and shear modulus were measured at the initiation of the first loading stage within a stress range between 0 and 2 MPa. The average Young’s moduli obtained in tension and compression were 2.7 GPa and 2.4 GPa, respectively, with standard deviations of 0.106 GPa and 0.080 GPa. This difference is evidence of asymmetry, and it may be due to the buckling of some polymer chains at very low compressive loads. Poisson’s ratios in tension and compression were 0.45 and 0.43, respectively. For an isotropic material, the shear modulus *G* can be deduced from Young’s modulus *E* and Poisson’s ratio ν by making use of the formula $G=\frac{E}{2(1+\nu )}$. The elastic properties in tension and compression for each sample yield a prediction of *G*; the average of these values in tension and compression were 0.94 GPa and 0.85 GPa, respectively. The average shear modulus measured directly in torsional tests was 0.97 GPa; this value is closer to the one deduced in tensile tests. Due to the asymmetry in the PC behavior, it is preferable to determine the shear modulus in pure shear stress state tests rather than deducing it from uniaxial tests.

Figure 4 and Figure 9 clearly reveal asymmetry in the material behavior. Positive plastic axial strains in both tension and compression are another proof of asymmetry. Moreover, in type 1 tests, the remaining axial and volumetric plastic strains in compression are approximately 40% smaller than those in tension.

### 4.3. Rejuvenation

Thermal rejuvenation eliminates the effects of physical aging and resets the thermal history, restoring the properties of a rejuvenated and freshly quenched polycarbonate. Thermal rejuvenation implies an increase in free volume, and since mechanical loads may leave positive volumetric strains, Struik and other authors have applied the term “mechanical rejuvenation” [11,39]. In ref. [40], McKenna questions the equivalence of thermal and mechanical rejuvenation after analyzing the differences between the effects of mechanical loads and thermal rejuvenation on some material properties, such as yield stress. Let us highlight additional differences between these processes that have not been previously published elsewhere. In a thermal rejuvenation of PC, the volume increase is isotropic, whereas the mechanical tests shown herein provoked an anisotropic volume increase: in tensile and compressive tests, positive plastic axial strains appeared without plastic transverse strains; in torsional tests, the tube thickness increased, but its length remained virtually the same. Thermal rejuvenation tends to reduce the elastic moduli of an aged polycarbonate; in contrast, the plastic volumetric strains did not affect Young’s modulus and shear modulus in tensile and torsional tests, respectively. We do not consider the term “mechanical rejuvenation” appropriate for the reasons above; in fact, as proposed by McKenna, a kind of phase transformation within the glassy state is more likely to occur.

### 4.4. Reasons for the Volumetric Strain Increase in Each Test

**Figure 15:**
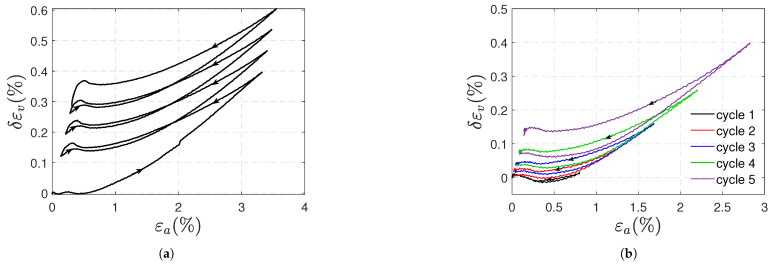
Volumetric strain $\delta \varepsilon_{v}$ (Equation (4)) vs. axial strain for type 1 (**a**) and type 2 (**b**) tensile tests (samples A).

For tensile tests, the volume increase can be explained by the fact that polymer chains experience localized stretching in the load direction, leading to nonlinearity and permanent strains after a significant chain alignment [41,42]. In order to determine the mechanisms underlying dilation in polymeric materials subjected to uniaxial tensile tests, Naqui and Robinson [43] proposed to calculate the difference $\delta \varepsilon_{v}$ between the measured volumetric strain $\varepsilon_{v}$ and the volumetric strain due to the dilational response $\varepsilon_{v}^{e}$, i.e., the volumetric strain that would be obtained if the material behaved elastically:

(4) $$\delta \varepsilon_{v}=\varepsilon_{v}-\varepsilon_{v}^{e}=\varepsilon_{v}-\left[\left(1+\varepsilon_{a}\right){\left(1-\nu \varepsilon_{a}\right)}^{2}-1\right],$$

where ν is the Poisson’s ratio, and $\varepsilon_{a}$ is the axial strain. According to Naqui and Robinson, if $\delta \varepsilon_{v}$ is positive, cavitation predominates as the deformation mechanism, and voiding contributes to an increase in the volumetric strains (positive plastic volumetric strains take place); otherwise, deviatoric mechanisms dominate, and shear banding occurs without an increase in the volumetric strain. The strains $\delta \varepsilon_{v}$ for samples A in type 1 and type 2 tests are plotted against the axial strains in Figure 15a,b, respectively; these strains are positive and increase with the load. It can be concluded that cavitation-type deformation mechanisms predominate under tension, resulting in an increase in volume and stretching of the polymer chains.

**Figure 16:**
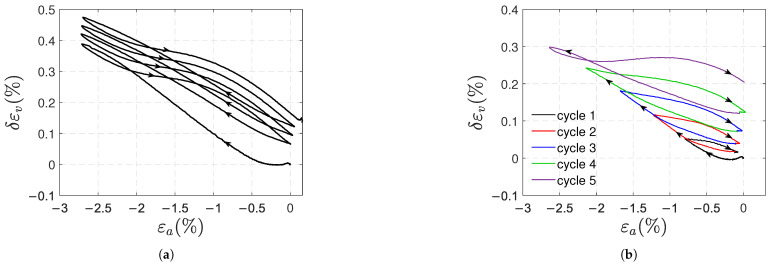
Volumetric strain $\delta \varepsilon_{v}$ (Equation (4)) vs. axial strain for type 1 (**a**) and type 2 (**b**) compressive tests (samples A).

In compressive tests, after removing the load, a positive plastic axial strain was measured, whilst the transverse strain was virtually zero. Therefore, a volume increase is also obtained. However, the increase in volume is more difficult to explain than for tensile results. According to Mulliken [42], compression provokes an equi-biaxial alignment in the transverse directions. In the axial direction, similar to what Drozdov [28] and Lai [41] suggested for the behavior of polymers in tension, it is suggested that the residual positive strains in compression are due to a localized rearrangement of the polymer chains. This rearrangement implies a disentanglement of the polymer chains, which requires more space and increases the free volume due to insufficient interchain packing. Let us extend the use of Equation (4) to compressive tests in order to attempt to identify the deformation mechanisms. For samples A, the curves of $\delta \varepsilon_{v}$ against $\varepsilon_{a}$ obtained in type 1 and type 2 tests are shown in Figure 16a and Figure 16b, respectively. The positive result may indicate that the deformation mechanism in compression is also of the cavitational type.

In torsion, in Figure 14a, the stress–strain curve did not reveal plastic shear strains. Still, the small hysteresis implies a dissipative mechanism: a fraction of the work conducted by external forces during a loading stage is not restored after the unloading one. This work fraction was dissipated and produced positive plastic strains across the thickness (along the first principal direction of the strain tensor). The tube length remained almost the same, as the other principal strains were virtually zero after unloading. A positive plastic volume strain is then obtained. This change in volume does not modify the shear response of the material: the stress–strain curves of each cycle are identical, as shown in Figure 14b. The stress state is purely deviatoric, contrary to what Naqui and Robinson stated in [43], and deviatoric mechanisms do indeed increase volume in a post-yield stage.

### 4.5. Are the Findings a Product of Experimental Flaws?

To ensure that the findings (hysteresis, expansion in compression, ratcheting, etc.) do not result from experimental errors or flaws in the design of the experiments and measurement devices, the following verifications were carried out:

- The critical buckling loads (in compression and torsion) are at least 1.8 times greater than the applied loads. Experimentally, in one of the compression tests, two axial strain gauges were placed—one on the front and one on the back of the specimen—and it was verified that there were no signs of bending, as both gauges measured virtually the same axial strain.
- The strain gauges accurately captured strain in cyclic tests. To confirm this, uniaxial cyclic tests were performed on steel specimens under loads that produced plastic strains of magnitudes similar to those presented here. The stress–strain curves did not exhibit ratcheting and showed that the unloading and subsequent reloading paths overlapped with no hysteresis (this is an expected result in steels subjected to small plastic deformations). Additionally, it was verified that the measured elastic modulus matched the typical value for steel.

It is worth mentioning that volumetric expansion in polymers under compression is not a novel observation. References [21,22,23,24,25,26] include works that report such behavior. However, it is common for these studies to report dilatation without verifying its directional nature. Moreover, this phenomenon is often attributed solely to the hydrostatic component of the stress tensor. Our results highlight that the deviatoric component also contributes to the volumetric deformation of such materials, as this component is present under uniaxial and shear loading conditions.

### 4.6. Does Plastic Expansion in Compressive Tests Happen Only in Polycarbonate Specimens?

**Figure 17:**
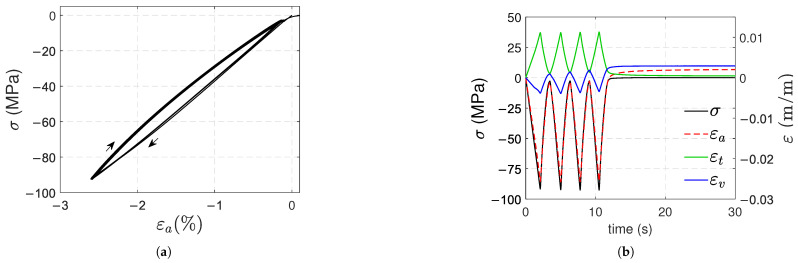
Stress vs. axial strain (**a**); stress and strains vs. time (**b**) curves for type 1 compression test with PMMA.

Although the results pertain to polycarbonate specimens, the observation of positive plastic axial and volumetric strains in compression appears to be a characteristic of the behavior of amorphous polymers in a glassy state. To verify this, a compressive test on a poly methyl methacrylate (PMMA) sample was performed. The dimensions of the sample were the same as those of the PC ones: 12 mm × 20 mm × 30 mm. A plate was made using a hot-plate press from material pellets to obtain this sample. A pressure of 1.9 MPa and a temperature of 160 °C were applied for 16 min. The specimen was thermally rejuvenated at 120 °C for four hours, then cooled to room temperature. Figure 17a displays the stress–strain curve obtained from the type 1 compressive test of the PMMA sample, exhibiting nonlinear behavior. Figure 17b shows the evolution of strains ($\varepsilon_{a}$, $\varepsilon_{t}$ and $\varepsilon_{v}$) and stress σ against time. The same phenomenon observed in the PC sample occurred in the PMMA sample: negligible plastic transverse strains were measured, but increasing positive plastic axial and volumetric strains were observed after each unloading stage.

## 5. Conclusions

In this paper, cyclic uniaxial and torsional tests were conducted on polycarbonate samples at room temperature. The strain rate used in the tests minimized the effects of viscosity. The following conclusions can be drawn:

- A nonlinear behavior was exhibited; it was caused by nonlinear elasticity and plasticity.
- Tensile and compressive tests revealed asymmetry in the material behavior in both pre-yield and post-yield states. Young’s moduli and Poisson’s ratios obtained in compression were slightly lower than those measured in tension.
- In tension, positive plastic axial and volumetric strains appeared, of course. Negative plastic transverse strains were obtained, but their magnitude was much lower than the plastic axial strain. The stress–strain curves exhibited ratcheting.
- In compression, positive plastic axial and volumetric strains also appeared; this was unexpected. Positive plastic transverse strains were measured, and their magnitude was once again marginal as compared to the plastic axial strain.
- In torsional tests with tubular specimens, a positive plastic volumetric strain also appeared. It was mainly due to the plastic strain across the thickness of the specimens.
- A deviatoric stress state does, in fact, provoke positive plastic volumetric strains.
- The concept of ”mechanical rejuvenation” is questionable in the tests performed herein; the volume increments in the post-yield stage were not uniform in all directions.
- Nonlinear elasticity was also revealed.

It appears that some of these conclusions can be extended to other glassy-state polymers: a preliminary compressive test on a PMMA sample also showed positive plastic axial and volumetric strains. These observations are crucial for a better understanding of the nonlinear behavior and volumetric changes in glassy-state polymers.

## Data Availability

Data is contained within the article.

## Abbreviations

The following abbreviations are used in this manuscript:

PC	polycarbonate
Tg	glass transition temperature
Mw	weight average molecular weight
PMMA	poly methyl methacrylate
